# Acute Balanitis Treated Locally with Ethyl Chloride

**Published:** 1904-07-23

**Authors:** 


### ACUTE BALANITIS TREATED LOCALLY WITH ETHYL CHLORIDE.

Vincent Hall [1] describes a case of retention of urine in a child resulting from balanitis. The child's condition would not allow of operation, and so he applied ethyl chloride in the form of a spray. The child ceased crying, the swelling subsided, and the child passed urine with comfort.

[1] The Mid. Med. Jour., July 1904.

